# The Use of the Veil by Operating Surgeons

**Published:** 1899-05-20

**Authors:** 


### THE USE OF THE VEIL BY OPERATING SURGEONS.

The recommendation made by Mikulicz, of Breslau, that surgeons should wear a veil of gauze or muslin while performing operations is a perfectly logical one, and quite in accordance with all that we know about the dissemination of infection, septic and other. The nose and mouth swarm with micro-organisms, and although these may be of an innocuous nature in persons who pass their lives in the open air in country places, it is often far otherwise with those who are exposed to many varieties of contagion, and, in view of the impossibility of disinfecting these cavities, it would seem almost absurd for those who hold to the necessity of using gloves to hesitate about taking to the veil when doing operative work. It may, no doubt, be maintained that expired air is practically germ free, and that if the operator will but keep silence there is no risk of infection reaching the wound from the mouth or nose. It is, however, a somewhat serious drawback not to be able to say a word throughout the length of an operation without turning the head on one side, and on the whole we think that the veil is likely to be found a distinctly useful appliance. What is certain is that ill-luck seems sometimes to dog the steps of some surgeons notwithstanding the most minute precautions in regard to aseptic technique, and it is by no means impossible that the personal factor in these unfortunate instances may have to do with the transport of infection by the breath. When we see the precautions taken in modern hospitals—the bath in the room adjoining the operating theatre ready for use by the surgeon before commencing his work, the division of the theatre itself by a glass partition, the sterilisation of instruments, dressings, water, hands, hair, and everything which comes near the patient, it clearly is ridiculous that the surgeon should keep blowing on to the seat of operation a stream of air coming from such septic cavities as the mouth and nose. The arguments in favour of the veil seem indeed unanswerable—unless we are prepared to admit that much else which is now the fashion in surgery is mere formality.

